# Reaction Kinetics and Process Model of the Polyacrylonitrile Fibers Stabilization Process Based on Dielectric Measurements

**DOI:** 10.3390/ma15031222

**Published:** 2022-02-06

**Authors:** Julia Hofele, Guido Link, John Jelonnek

**Affiliations:** 1 Institute for Pulsed Power and Microwave Technology (IHM), Karlsruhe Institute of Technology (KIT), 76131 Karlsruhe, Germany; Guido.Link@kit.edu (G.L.); John.Jelonnek@kit.edu (J.J.); 2 Institute of Radio Frequency Engineering and Electronics (IHE), Karlsruhe Institute of Technology (KIT), 76131 Karlsruhe, Germany

**Keywords:** carbon fiber, dielectric properties, reaction kinetics, stabilization, microwave heating, heat transfer, modelling

## Abstract

Microwave-based dielectric heating is a suitable method for energy- and time-efficient processes. Considering the energy required in the production of carbon fibers, it is evident that microwave-based dielectric heating during the different phases of the production needs to be considered too. Nevertheless, the dielectric properties of the processed material needs to be known for the design of an appropriate microwave applicator. When looking at the first stage in the production, the stabilization stage of the PAN fiber, the important data about the dielectric properties is very limited in literature. For this reason, first in-situ temperature-dependent measurements of the dielectric properties during the stabilization stage are presented. The impact of raising temperatures and chemical reactions on the dielectric properties of the heated PAN fiber is discussed. Secondly, the steps taken to set up the reaction kinetics from the dielectric loss point of view are given. This enables determination of the reaction degree as a function of the measured dielectric loss for the first time. The established correlation opens the potential for the application to processes such as an in-situ quality determination. The strong temperature impact on the process is shown, and reaction kinetics are analyzed accordingly. In a final third step, a heat transfer model is presented. It utilizes the evaluated reaction kinetics data and microwave heating, creating a first modelling approach for monitoring and controlling the desired fiber temperature, leading towards an online process.

## 1. Introduction

**Figure 1:**
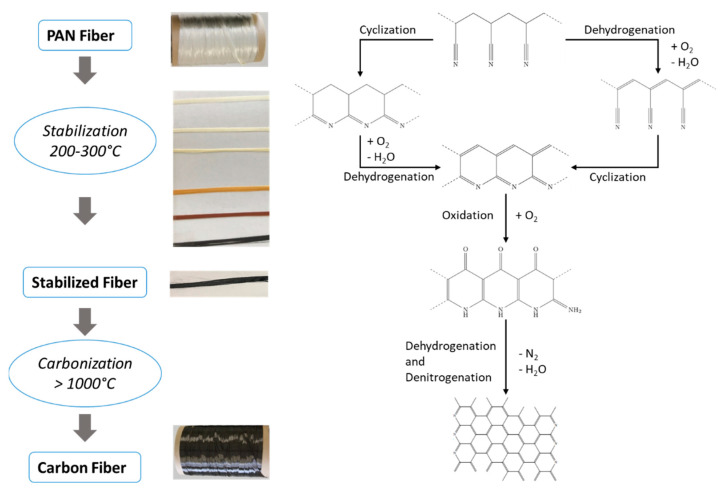
Reaction schematic of the stabilization stage.

Carbon fibers are used in multiple lightweight applications due to their good physical properties [1]. Their tensile strength to density ratio is superior to other materials such as aluminum or steel. However, carbon fiber production needs significantly more energy than production of aluminum [2]. Until today, carbon fibers are typically produced in conventional convection ovens. Different precursor materials are used to produce carbon fibers with Polyacrylonitrile (PAN) being the most commonly used one. The carbon fiber manufacturing process steps are listed in Figure 1 on the left side. A spun PAN fiber gets stabilized in normal air and using a conventional oven at temperatures ranging from 200 °C up to 300 °C as a first step in the production process. The total process time can take up to several hours depending on the temperature steps applied, the diameter of the precursor and the chemical composition of the PAN fiber. Chemical transformations through oxidation, cyclization and dehydrogenation affect the fiber in the stabilization stage. The fiber color changes from white to light brown, to dark brown and ultimately to black. The former linear chemical structure of the fiber transforms into an inflammable cyclic ladder polymer (Figure 1 right side) [1,3]. All three reactions are exothermic, adding additional heat to the fiber, which has to be considered when optimizing the stabilization process. 

In a second step, the fiber is heated above 1000 °C in an inert gas atmosphere, often made of nitrogen or argon. Specific time and temperature conditions are chosen depending on the desired final fiber grade. In this phase of production, the fiber is restructured internally. The ladder polymers are cross-linked, and nitrogen is removed from the aromatic rings. The carbon fiber acquires electrically conductive properties [1]. Sometimes an additional step, post-processing the fiber, is mentioned in the fiber production. The post-processing step prepares the fiber surface for interaction with a resin necessary to form carbon fiber composites. The surface is roughened up and/or is covered with a sizing agent to improve the adhesion of a resin to the fiber [1].

To shorten the process time and, even more importantly, to increase the process and energy efficiency, different approaches are currently investigated, including optimization of heat distribution and insulation of conventional ovens. Alternatively, as a radically different approach, the replacement of the conventional heating with plasma and microwave heating respectively in at least one of the process steps is explored as well. Considering dielectric heating using microwaves offers volumetric and selective heating. Additionally, it allows fast-response temperature control. The knowledge about the dielectric loss $\epsilon_{r}^{\prime \prime }$ of the material is key for the design of an appropriate applicator.

Focusing only on the stabilization stage, the below patents and publications describe improvements for the stabilization process using microwaves and microwave plasma.

In 2004, Paulauskas and White [4] published the dielectric properties of PAN fibers, but no information about the final stabilization degree was provided. Moreover, a holding time was neither mentioned nor used, and the cooling down process was not measured. In [5], Chao et al. provide a dielectric characterization of the PAN fibers after each processing stage. The properties of the given samples are evaluated at room temperature only. As the measurements at different temperatures are important in order to understand the chemical influence on the dielectric properties during the chemical transformation, this means another gap of information. For example, the start of the reactions is influenced by using different copolymers in the PAN fiber [6].

The patent EP2475812B1 [7] by Toho Tenax Europe GmbH outlines a microwave applicator which uses a combination of a preheated process gas of 150 to 300 °C and microwave heating to stabilize the fiber. The microwave applicator uses areas of different electric field strengths, going from 3 to 150 kV/m. Different applicator examples are presented, ranging from a rectangular waveguide of 1 to 1.5 m length. In US7786253B2 [8], and US20150252501A1 [9] by UT-BATTELLE, LLC, Oak Ridge, an apparatus using non-thermal atmospheric pressure plasma to stabilize the fibers is used. They use reactive oxidative species, such as monoatomic oxygen, to increase the speed of oxidation. Patent US6375875 [10] describes a method for monitoring the dielectric properties of the fiber during all carbon fiber production steps. In 2004, Paulauskas et al. used atmospheric pressure plasma to stabilize PAN fibers [11]. Different process gases were applied. It was discovered that the plasma treatment leads to a more homogeneous oxidized cross section of the fiber while resulting in lower densities. The plasma stabilization reduced the processing time by a factor of three compared to conventional heating, with a line speed of 0.3 m/min and less than 35 min of total processing time.

In 2011 and 2013 Lee et. al. [12,13] showed the influence of plasma treatment in the stabilization process on the fiber. Due to the plasma treatment, atomic oxygen was concluded to be predominant in the process atmosphere. As atomic oxygen is smaller than diatomic oxygen, it diffuses faster into the fiber filaments and can thus shorten the oxidation process. Furthermore, plasma stabilization did result in higher tensile strength of the fibers, compared to the conventional process. The extent of reaction was defined according to the degree of cyclization. A 15 min plasma treated fiber showed the same degree of cyclization like the conventionally treated fiber after 120 min.

Dielectric heating was investigated in 2018 by Liu et al. [14] and Zhang et al. [15]. Both compared it to conventionally heated fibers. Liu et al. suggested the skin-core structure is less distinctive due to the volumetric heating effect of the microwaves [14]. Microwave heating shortened the process time by 35 min and reduced the process temperature by 30 °C. However, the fiber was tied to a ceramic rod, and no dielectric properties were provided. Moreover, neither the location nor the method for the fiber temperature measurement is mentioned. In [15] it is stated that microwave heating shortens reaction time and improves the fiber surface. Again, the fibers are attached to a ceramic rod, and no dielectric properties are provided. It can be assumed that the microwave was predominantly heating the ceramic rod at the beginning, and the fiber is heated via thermal conduction. Only later in the process, when dielectric loss of the fibers is increasing with temperature, to some extent a direct microwave heating effect might take place. But as no dielectric properties are provided, it is not possible to definitely draw this conclusion. In [16], Zhao et al. investigate the influence of microwave oxidation on the reaction mechanisms and the fiber structure. They describe an improvement of the structure caused by reduced surface defects.

Another option to improve the quality of the stabilized fibers is the application of appropriate theoretical models for the stabilization process. A first heat transfer model for the fiber temperature was created by Dunham and Edie [17] in 1991. An overview of published models, such as reaction kinetic and heat transfer models, can be found in [18]. Terra et al. [19] used intelligent algorithms for modelling the stabilization stage. The target was analyzing the influence of several process parameters on the quality of the fiber. The authors stated the volumetric density and a FTIR (Fourier-transform infrared, Thermo Scientific 4700 Nicolet, Thermo Fisher Scientific Inc.) conversion index may be suitable tools for control of the fiber quality during the stabilization stage.

In general, different tools are available for the evaluation of the final carbon fiber quality [6]. As the quality after stabilization has a strong influence on the final fiber quality, evaluation of the fiber quality is already of interest after the stabilization. Often a stabilization degree is defined and measured, for example with FTIR spectroscopy (Nicolet iS50, Nicolet Fisher, MA. USA) [16] or DSC (Differential scanning calorimetry, Mettler Toledo) measurements [19]. Yu et al. [20] provide a short overview of various methods and use of X-ray diffraction for characterizing the stabilization degree. So far, dielectric properties are not used as standard for determining the fiber quality. Even though some measurements of the dielectric properties of PAN fibers were already provided, thorough data is not available. Moreover, the chemical composition may affect the dielectric behavior.

For designing a suitable microwave applicator, it is key to understand the dielectric properties of the material depending on chemical composition, temperature and chemical reaction. For this reason, dielectric measurements are presented first, followed by an investigation of the reaction kinetics in the stabilization stage from a dielectric point of view. Knowledge about the reaction kinetics results for the first time in the possibility to determine the stabilization degree, and therefore to which amount the reaction took place, based on the dielectric loss. The link between dielectric loss and degree of stabilization can be used for online monitoring of the fiber quality. In a third step, the obtained link allowed to generate a mathematical model of the temperature behavior during the stabilization stage with microwave heating. To the knowledge of the authors, so far microwave heating has not been included in the modelling of the stabilization process, and no connection between reaction kinetics and dielectric properties was made. Adding the microwave heating in calculating the temperature development can help understanding and controlling the process. Furthermore, it and may help in design and development of appropriate microwave applicators and optimize process parameters that allow avoiding process instabilities due to thermal runaway effects.

## 2. Materials and Methods

### 2.1. Materials

The fibers used here are composed of more than 95% Polyacrylonitrile with a 5% mixture of Methyl acrylate (MA) and Itaconic acid (IA). The fiber bundle used consists of 12,000 filaments (12k).

### 2.2. Dielectric Measurements with the Perturbation Method

For dielectric measurements, often the classic perturbation method is used. Resonance frequency and quality factor of the empty system are measured, then the sample is inserted into the cavity. This leads to a perturbation of the electric field, resulting in a shift of resonance frequency and quality factor. Ideally, the sample is placed in the electric field maximum to get the strongest effect. The shift is measured using a network analyzer. The perturbation method applies the assumption that a small sample with medium or low losses is not significantly altering the electrical field and thus the mode. With good approximation the dielectric properties can then be calculated from the following equations [21]:

(1) $$\epsilon_{r}^{\prime }=\frac{1}{A_{cal}}\frac{f_{\mathrm{ref}}-f_{s}}{f_{s}}\frac{V_{c}}{V_{s}}+1$$

(2) $$\epsilon_{r}^{\prime \prime }=\frac{1}{B_{cal}}\left(\frac{1}{Q_{s}}-\frac{1}{Q_{\mathrm{ref}}}\right)\frac{V_{c}}{V_{s}}$$

where $\epsilon_{r}^{\prime }$ is the relative dielectric constant, $\epsilon_{r}^{\prime \prime }$ is the relative dielectric loss, $f_{\mathrm{ref}}$ and $f_{s}$ are the resonance frequencies of the unperturbed (empty) and perturbed (with sample) cavity, respectively. $Q_{\mathrm{ref}}$ and $Q_{s}$ are the corresponding quality factors. $V_{c}$ and $V_{s}$ are the volumes of the cavity and the sample. The calibration factors $A_{cal}$ and $B_{cal}$ depend on the sample and cavity geometries, the coupling, and on the final cavity wave pattern of the mode. An adapted version of the perturbation method was used in order to improve the accuracy, as in practice $A_{cal}$ and $B_{cal}$ are dependent on the permittivity [22]. A full wave simulation of the setup was performed using the software CST Microwave Studio (CST Studio Suite, Dassault Systèmes, Vélizy-Villacoublay, France) [23] within the range of the expected dielectric properties to calibrate factors $A_{cal}$ and $B_{cal}$. The CST library materials were used except for the sample material. The Eigenmode Solver was used for obtaining the resonance frequency and quality factor of the system.

**Figure 2:**
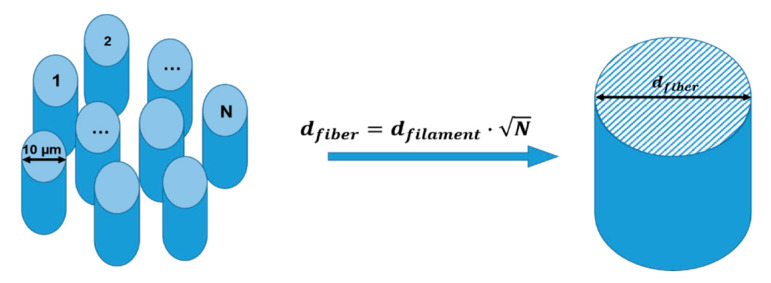
Simplification of the fiber in the 3D electromagnetic simulation by a cylinder with an effective fiber diameter.

For evaluation of the dielectric properties and to set up the calibration with the model, the sample volume needs to be determined. The fiber consists of a large number of filaments, in the order of several thousands. With a filament diameter of about 10 µm each, a suitable approximation must be made to perform a 3D electromagnetic simulation. Here, it is proposed to replace the large number of individual filaments by a single cylinder with an effective fiber diameter as shown in Figure 2. The fiber diameter can be either calculated from the filament diameter or with the help of density and mass measurements of a fiber bundle with a given length.

**Figure 3:**
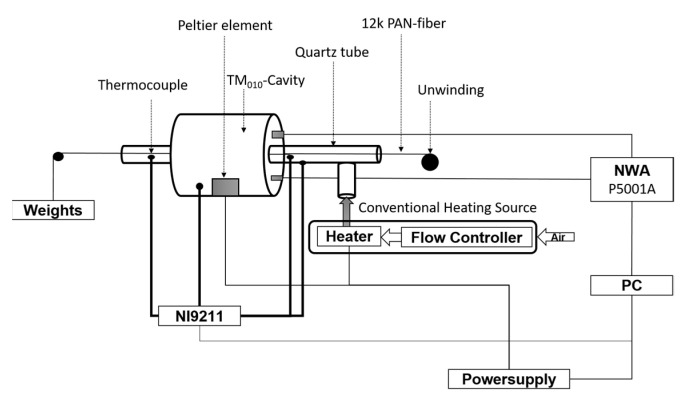
Schematic drawing of in-situ measurement setup of the temperature-dependent dielectric properties.

A schematic drawing of the measurement setup used is shown in Figure 3. The measurement is performed in a cylindrical single mode TM_010_-mode cavity made from copper with a resonance frequency of about 2.45 GHz and a quality factor for the empty cavity of about 10,000. Cavity size, both in height and radius, was designed to be 45 mm. The ratio of radius to height is chosen to avoid the appearance of unwanted modes close to the resonant frequency [24]. The precise dimensions of the manufactured cavity were evaluated by measuring the frequency of the TM_010_ and TE_111_ modes. An N-Type port with a pin antenna is used for input, and a SMA (SubMiniature Version A) port, also equipped with a pin antenna, is used for output. Coupling of the electrical signal can be adjusted through the length of the pin antennas. This also influences the overall quality factor of the system. The final coupling was evaluated based on the measured S-Parameters and resulted in pin lengths inside the cavity of 3.1 mm for the N-Type port and 1.8 mm for the SMA port. The 12k PAN fiber is located inside a quartz tube and placed along the maximum electric field of the TM_010_-mode. To obtain a defined fiber tension, the fiber is fixed resulting in batch processing. The quartz tube position in the microwave cavity is fixed by using Teflon insulation ring. A Keysight P5001A (Keysight Technologies Deutschland GmbH, Böblingen, Germany) Vector Network Analyzer (VNA) measures the resonance frequency and quality factor of the system. The power level is kept at 0 dBm output power. For the temperature dependent measurements, a 1600 W heater, type MK-45R, from Zinser GmbH (Albershausen, Germany) is used. To control the hot air temperature the heater has been modified using a thyristor controller from A-senco type SCR-800 (Pohltechnic.com GbR, Aalen, Germany) together with an analog power control. A mass flow controller, type GSC-D4SA-BB12 (Vögtlin Instruments GmbH, Muttenz, Switzerland), was used to stabilize the input air flow rate to 100 ln/min. The air is heated up as it passes the heating cartridge and is channeled into the quartz tube where it heats the PAN fiber. A NI-9211 thermocouple (National Instruments, Austin, TX, USA) input module is installed to measure the temperature using type K thermocouples with an accuracy of ±2.2 °C and a measurement sensitivity of 0.07 °C [25]. Two thermocouples are placed at the entry and the exit point of the cavity inside the quartz tube to measure the air flow temperature. A third one is used to measure the quartz tube outer surface temperature, and a fourth one for the cavity temperature monitoring. Using a 180 W Peltier element the cavity temperature can be stabilized to a preset value of typically 20 °C with an oscillation of ±0.3 °C.

### 2.3. Dielectric Measurements Error Evaluation

The accuracy of the measured dielectric properties is influenced primarily by the following main parameters: temperatures of the cavity and the quartz tube; positioning of the quartz tube and the sample; and the sample volume used in the calibration. Other influencing factors include the accuracy of the VNA measurement and the accuracy of the applied simulation. Since the shift of the quality factor with the PAN fiber is rather small at room temperature, measurement precision strongly depends on the accuracy of the resonance frequency measured, and the quality factor of the empty cavity. Ensuring a reproducible reference can be significantly improved by stabilizing the cavity temperature. A temperature of 20 °C ± 0.3 °C limits the frequency shift due to thermal expansion of the cavity to below 11.6 kHz. A change in quartz temperature leads to a frequency shift as well, whereas the quality factor is not affected by a temperature change of the quartz tube in the given process parameter range. An increase of the temperature of the air inside the quartz tube up to 235 °C leads to a shift in the resonance frequency of about 275 kHz. Thus, to distinguish the effect of the PAN fibers from the effect of the quartz tube, the temperature-dependent frequency shift of the stabilized cavity with an empty quartz tube is measured separately for each set of process parameters and used as reference. The impact of the quartz tube position is minimized by using the junction of the quartz tube as a stopping mechanism when inserted into the cavity. The Teflon insulation ring ensures to keep the tube at fixed position in the field maximum and provides a thermal insulation. The overall error calculation is demonstrated by calculating the error of the dielectric loss $\sigma_{\epsilon_{r}^{\prime \prime }}$. Describing the shift of the quality factor $D_{Q}$ according to Equation (3) leads to the error of the dielectric loss $\sigma_{\epsilon_{r}^{\prime \prime }}$ in Equation (4) [26]. Here, σ describes the error of the respective indices introduced in Equation (2). The simulation error $\sigma_{B}$ depends on mesh size and simulation accuracy. Those parameters were chosen to be fine enough to exclude any influence on the simulation result. The error from the sample volume $\sigma_{V_{s}}$ was determined by using a mean value of the effective fiber radius. This dimension got evaluated by multiple weight measurements, length, density of the bundle, and filament diameter measurements in a microscope. The mean value of the effective fiber bundle radius resulted to 0.54 mm ± 0.01 mm. The dielectric loss can be evaluated with a measurement error of around ±1%.

(3) $$D_{Q}=\frac{1}{Q_{s}}-\frac{1}{Q_{\mathrm{ref}}}$$

(4) $$\sigma_{\epsilon_{r}^{\prime \prime }}=\sqrt{{\left(\frac{\partial \epsilon_{r}^{\prime \prime }}{\partial D_{Q}}\sigma_{D_{Q}}\right)}^{2}+{\left(\frac{\partial \epsilon_{r}^{\prime \prime }}{\partial V_{s}}\sigma_{V_{s}}\right)}^{2}+{\left(\frac{\partial \epsilon_{r}^{\prime \prime }}{\partial V_{C}}\sigma_{V_{C}}\right)}^{2}+{\left(\frac{\partial \epsilon_{r}^{\prime \prime }}{\partial B}\sigma_{B}\right)}^{2}}$$

### 2.4. Thermal Model

A thermal model is the first step towards online monitoring and controlling of a continuous process with microwave heating. It helps building an understanding of the influence of the process parameters and thus can help to predefine the range in which the input variables lead to the desired output temperature instead of a thermal runaway. The model calculates the temperature rise induced by the microwaves, the time inside the microwave system, the reaction heat from the exothermic reactions, and the heat transfer. The temperature at every z-position along the cavity length can be calculated and thus can be used to predict the behavior of the fiber during the reaction.

**Figure 4:**
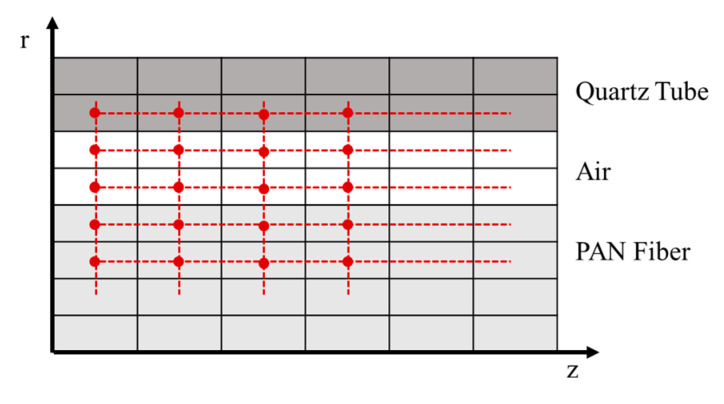
2D-schematic sketch of the mesh cells in the thermal model.

As the setup is symmetrical, a 2D model is sufficient to simulate the PAN fiber mounted in the quartz tube. The 2D model comprises multiple mesh cells in axial and radial direction. The number of mesh cells can be varied in both directions. This enables the user finding the optimum of computation time versus result accuracy. A mesh example is shown in Figure 4, where the fiber is modeled with four cells in radial direction, followed by two cells for the air flow and two cells for the given quartz tube. Each cell is connected to the adjacent cells in axial and radial direction through the thermal equations. The Finite Volume Method (FVM) is implemented using MATLAB (MATLAB Version 2019b, Mathworks^®^, Natick, MA, USA) [27] to solve the thermal equations for each cell.

The calculation of the temperature change for each element is given by the equation:

(5) $$\frac{\partial T}{\partial t}=\frac{{\dot{Q}}_{reac}+{\dot{Q}}_{conv}+{\dot{Q}}_{cond}+{\dot{Q}}_{rad}+p_{V}\cdot V_{cell}}{m_{cell}\cdot c_{p}}$$

where T is the temperature in K, t the time, the heat flow produced by the reactions ${\dot{Q}}_{reac}$, the convection ${\dot{Q}}_{conv}$, the conduction ${\dot{Q}}_{cond}$, and the radiation ${\dot{Q}}_{rad}$ inside each cell, $p_{V}$ the power density based on microwave absorption, $V_{cell}$ the volume of the current cell element, $m_{cell}$ the mass of the cell and $c_{p}$ the specific heat capacity. The different heat flows are given as follows [28]:

(6) $${\dot{Q}}_{conv}=\alpha_{th}\cdot A_{S}\cdot \left(T_{1}-T_{2}\right)$$

(7) $${\dot{Q}}_{cond}=\lambda_{th}\cdot 2\cdot \pi \cdot L\cdot \frac{\left(T_{1}-T_{2}\right)}{\ln\left(\frac{r_{o}}{r_{i}}\right)}\;$$

(8) $${\dot{Q}}_{rad}=\frac{\sigma \cdot A_{S,1}\cdot (T_{1}^{4}-T_{2}^{4})}{\frac{1}{\varepsilon_{1}}+\frac{A_{S,1}}{A_{S,2}}\left(\frac{1}{\varepsilon_{2}}-1\right)}$$

where $\alpha_{th}$ is the heat transfer coefficient of the material, $A_{S}$ the surface area, $\lambda_{th}$ the thermal conductivity, L the length, $r_{o}$ and $r_{i}$ the outer and inner radius, respectively.

In Equation (8) σ refers to the Stefan–Boltzmann constant, $\varepsilon_{1}$ and $\varepsilon_{2}$ to the emission coefficient of the respective material, and $A_{S,1}$ and $A_{S,2}$ to each surface area.

The heat flow obtained through the chemical reactions ${\dot{Q}}_{reac}$ is based on the reaction kinetics evaluated from the dielectric loss measurements in combination with the heat of reaction evaluated for two reactions, the cyclization and the oxidation enthalpies.

The power density $p_{V}$ absorbed inside a dielectric material from microwave heating can be calculated from the equation [29]:

(9) $$p_{V}=2\pi f\epsilon_{0}\epsilon_{r}^{\prime \prime }E^{2}$$

where f is the microwave frequency, and $\epsilon_{0}$ is the vacuum permittivity. E is the amplitude of the electric field.

## 3. Results

**Figure 5:**
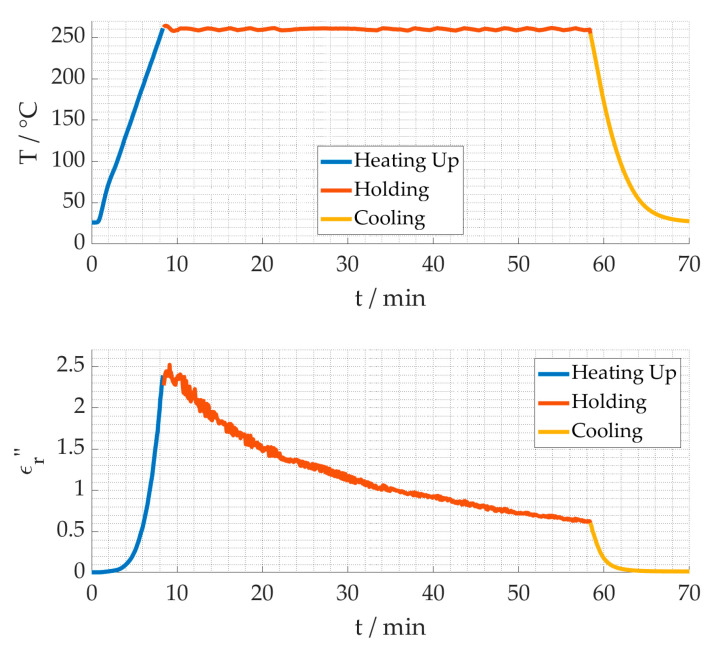
Dielectric loss for a process time of 50 min and at a process temperature of 260 °C.

In this section, temperature-dependent dielectric measurements are presented. The temperature profiles include a heating rate of 30 °C/min, a varying holding time at the process temperature and subsequent free cooling. The temperature, resonance frequency and quality factor are measured every few seconds by use of a MATLAB based process control and data acquisition program. In Figure 5 the change of the dielectric loss during the heating up (blue), holding (red) and cooling down (yellow) phase is shown for a process temperature of 260 °C and a holding time of 50 min. The results show the dielectric loss increases strongly from a low loss starting value of 0.007 to 2.5 at higher temperatures (blue). It decreases when the temperature is kept constant (red) to a value of around 0.6 during isothermal processing at 260 °C. When cooled down (yellow), the dielectric loss decreases even further to 0.016, but remains higher than the starting value. The changes occurring during the isothermal processing and the higher end value after processing lead to the conclusion that the chemical reaction was taking place.

The loss factor $\epsilon_{r}^{\prime \prime }$ is measured with an accuracy of ±0.0017 for the virgin PAN fiber at room temperature. The worst case for the accuracy of the heated fiber is ±0.14. It is caused by a decrease in the sample quality factor $Q_{s}$ resulting in an increase of Equation (3). The stabilized fiber is measured at room temperature with an accuracy of ±0.0019.

**Figure 6:**
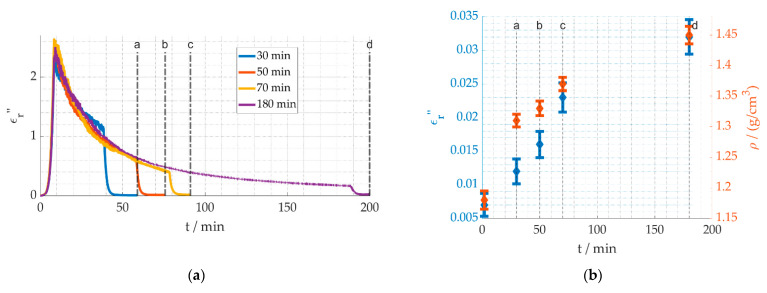
Dielectric loss and densities for different process times with a process temperature of 260 °C (Adapted from [31]). (**a**) Time dependent change of the dielectric loss for different process times. (**b**) Dielectric loss and density at room temperature after the process.

In Figure 6a, the permittivity versus processing time at a process temperature of 260 °C is shown. The data demonstrate that for longer holding times, the chemical reaction is still progressing. Even after 180 min at 260 °C, a small change in the dielectric loss is still measurable. Figure 6b compares the dielectric loss and densities of the fibers that have been processed according to Figure 6a after the process has been set back to room temperature. The compared values are marked with a, b, c and d. The corresponding material properties of the unprocessed fiber are included at 0 min. The longer the processing time, the more the dielectric loss is increased back at room temperature, also displayed in the density increase. Therefore, the density can be used as an indication for the stabilization degree, as can the dielectric losses. Values for stabilized industrial PAN fibers are in the range of 1.37 to 1.39 g/cm^3^ [30]. Similar density results are achieved for a holding time of 70 min (see c). The different processing times can be clearly distinguished in the dielectric loss. This allows for the next step, linking the dielectric loss to the reaction kinetics.

**Figure 7:**
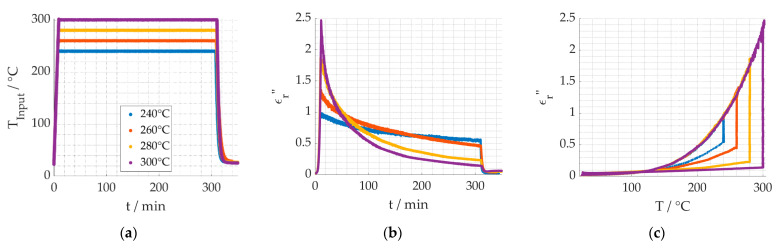
Dielectric loss long term measurement for different temperatures. (**a**) Temperature profiles. (**b**) Dielectric loss over time. (**c**) Dielectric loss over temperature.

The first step towards understanding the reaction kinetics is gathering long term data of the reaction. In Figure 7a, the temperature profiles are shown. The process temperatures 240 °C, 260 °C, 280 °C and, ultimately, 300 °C were used for a holding time of 300 min. Figure 7b shows temperatures are strongly affecting the slope of the decreasing dielectric loss, when the temperature is kept constant, thus indicating a higher reaction speed. Figure 7c shows after holding the temperatures for 300 min the dielectric loss is still higher at lower process temperatures, demonstrating the reaction progress is less than for higher temperatures. It is assumed that the cyclization and dehydrogenation reactions are completed and the stabilization degree for them is 100%.

## 4. Discussion

### 4.1. Reaction Kinetics

For the next step towards determining the reaction kinetics, some important fundamental equations are presented first [32]. In general, the degree of conversion, hence the reaction progress α can be described as

(10) $$\frac{d\alpha }{dt}=k\left(T\right)\cdot f\left(\alpha \right)$$

where f(α) is a function of the degree of conversion and k(T) is the Arrhenius equation expressing the temperature dependency of a reaction as

(11) $$k\left(T\right)=k_{0}\cdot e^{-\frac{E_{a}}{R\cdot T}}$$

with $k_{0}$ is the pre-exponential factor, $E_{a}$ is the activation energy, R is the universal gas constant, and T stands for the absolute temperature. The simplest assumption for f(α) is

(12) f(α)=1−α

Under the assumption that the temperature of the process is constant, the Equation (10) with Equation (12) can be transformed into

(13) $$\alpha =1-e^{-t\cdot k\left(T\right)}$$

In the case of the stabilization, multiple chemical reactions take place. Separating the reactions is not possible as the dehydrogenation and the oxidation both require oxygen to take place and the cyclization and the dehydrogenation are taking place at the same time. The cyclization can be singled out from the other reactions if an inert gas atmosphere is used. Nevertheless, in a first simplified approach, it was chosen to use two reactions that follow after each other in the reactions model, where the first reaction comprised both the cyclization and dehydrogenation. Data from literature suggest similar activation energies for both reactions, so that the influence of combining them is assumed to be negligible [33].

**Figure 8:**
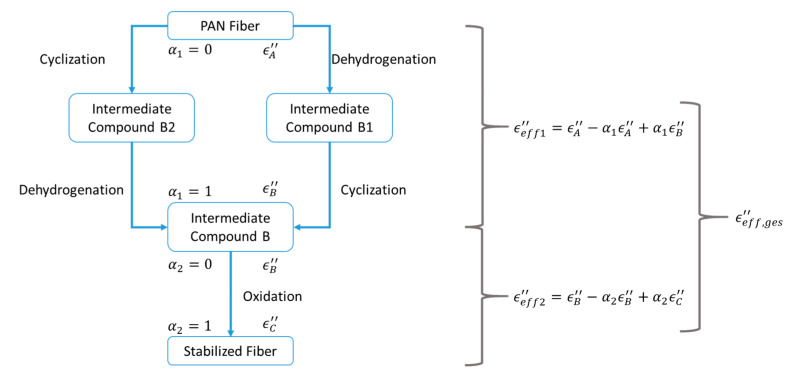
Diagram showing linkage between degree of conversion and dielectric loss during the stabilization stage.

Figure 8 shows a schematic sketch of the steps taken to link the dielectric loss to the degree of conversion. Generally speaking, the effective dielectric loss $\epsilon_{eff}^{\prime \prime }$ can be connected to the degree of conversion of a reaction α by use of an appropriate mixing relation. A linear connection between the start and end material was chosen as basic approach, see Equation (14). It is dependent on the degree of conversion α, the dielectric loss of the material before the reaction took place, labeled with $\epsilon_{Start}^{\prime \prime }$, thus the starting material and the dielectric loss of the material after the reaction, labeled with $\epsilon_{End}^{\prime \prime }$, thus the transformed end material:

(14) $$\epsilon_{eff}^{\prime \prime }=\left(1-\alpha \right)\cdot \epsilon_{Start}^{\prime \prime }+\alpha \cdot \epsilon_{End}^{\prime \prime }$$

In the case of the stabilization, the intermediate product after cyclization and dehydrogenation was chosen as the end product to describe the first reaction, while the stabilized fiber was chosen as the end product of the second reaction. Both effective dielectric losses were then combined into one overall effective dielectric loss scheme.

Thus Equation (14) was applied for both reactions, see also Figure 8 $\epsilon_{eff,1}^{\prime \prime }$ and $\epsilon_{eff,2}^{\prime \prime }$, resulting in the final equation for the overall dielectric loss

(15) $$\epsilon_{eff,ges}^{\prime \prime }=\left(1-\alpha_{1}\right)\epsilon_{A}^{\prime \prime }+\alpha_{1}\left(1-\alpha_{2}\right)\epsilon_{B}^{\prime \prime }+\alpha_{1}\alpha_{2}\epsilon_{C}^{\prime \prime }$$

With Equation (13) the effective dielectric loss for the complete reaction leads to

(16) $$\epsilon_{eff,ges}^{\prime \prime }=(\epsilon_{A}^{\prime \prime }-\epsilon_{C}^{\prime \prime })\cdot e^{-t\cdot k_{1}}+(\epsilon_{B}^{\prime \prime }-\epsilon_{C}^{\prime \prime })\cdot e^{t\cdot k_{2}}+(\epsilon_{C}^{\prime \prime }-\epsilon_{B}^{\prime \prime })\cdot e^{-t\cdot (k_{1}+k_{2})}+\epsilon_{C}^{\prime \prime }$$

As the dielectric properties show an exponential temperature dependency—as shown in Figure 7c—both for the heating up and the cooling down phase, the following equation is used to describe the temperature dependent dielectric losses of the compounds A, B and C, according to Figure 8, assuming no reaction is taking place:

(17) $$\epsilon_{A,B,C}^{\prime \prime }=c_{A,B,C}\cdot e^{-\frac{b_{A,B,C}}{T}}$$

where $c_{A,B,C}$ and $b_{A,B,C}$ are constants.

The next step is to use the software MATLAB [29] and its fitting toolbox to fit Equation (16) under consideration of Equations (11) and (17) to the measured data of Figure 7. The result is shown in Figure 8, and the fitted values are listed in Table 1, with a residual norm of 69. The 95% confidence intervals for the fitting parameters were obtained using the “nlparci” function of MATLAB.

**Figure 9:**
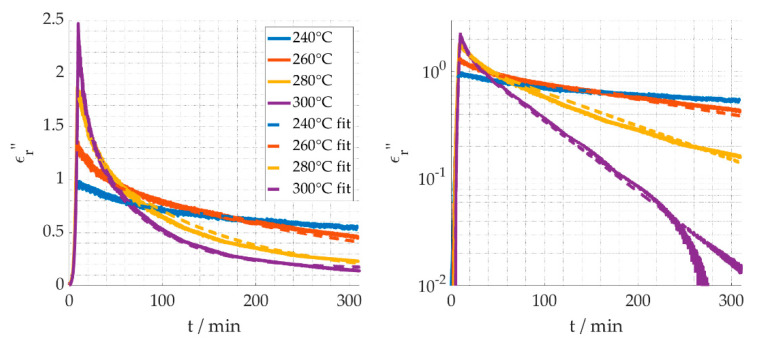
Linear (**left**) and logarithmic (**right**) graphical presentation of the measured dielectric loss and the fitted curves.

The fitted values are reasonably closely correlated to the measured values. Moreover, the activation energies are in agreement with values from the literature, i.e., as provided by Badii et al. [34]. The cyclization activation energy is given with 81.6 kJ/mol, and 40.9 kJ/mol for the reduction reaction. Badii adds the reduction parallel to the oxidation to the stabilization mechanism. The deviation of the 300 °C curve in the logarithmic presentation (Figure 9, right) is caused by the difference of the calculated versus the actual limiting value $\epsilon_{C}^{\prime \prime }$. Similarly, the slight noise in the fit curves is caused by using the measured temperature profiles.

**Figure 10:**
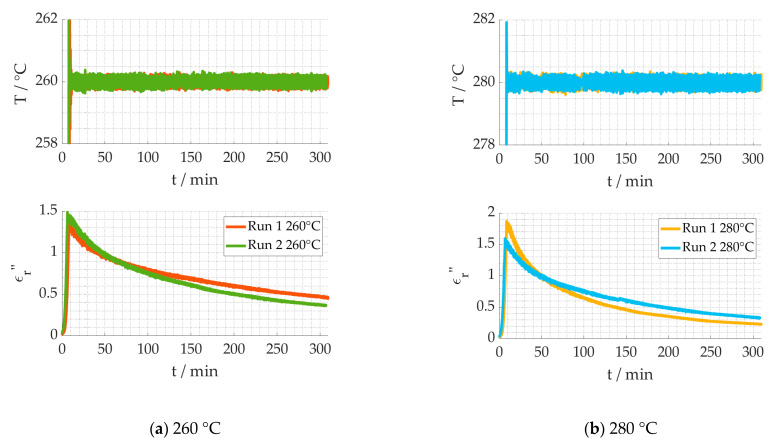
Dielectric loss of the same process parameters measured at different campaigns.

The stabilization process is, amongst other things, dependent on the process temperature. In conventional ovens, the process temperature is strongly influenced by the temperature of the surrounding air mass, while the exothermic reactions are adding extra energy. In the experimental setup, the air temperature was controlled by a thermocouple, but the reproducibility of the thermocouple placement as well as the control loop and the exothermic reactions may cause day-to-day variations of the actual temperatures. These variations of the dielectric loss from different measurements, while using the same process parameters, called “Run”, are shown in Figure 10. Run 1 refers to the same data as in Figure 7 and Figure 9 of the same temperature (same color). The temperature data shows that the measured temperature was almost identical, while the dielectric behavior varies over time. Thus, it is evident that the kinetic parameters evaluated for Run 1 will not lead to a good fit with the data of Run 2. The assumption can be made that the effective temperature in each Run is different.

**Figure 11:**
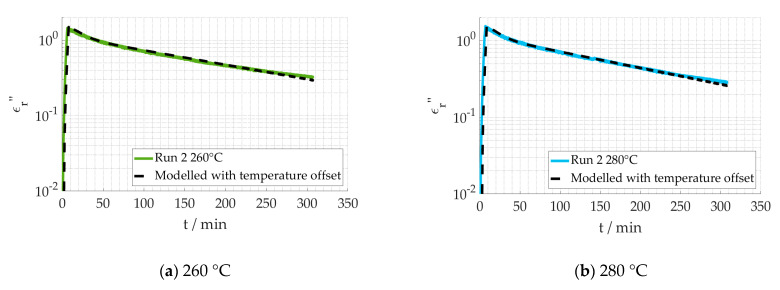
Logarithmical comparison between measured dielectric loss and fitted values using a temperature offset.

For the benefit of improving the fit and validate the assumption, a new parameter is introduced, adding a temperature offset to the measured temperature. The resulting fit for Run 2 of 260 °C and 280 °C can is shown in Figure 11. According to the dielectric behavior measured, the effective temperature of the 260 °C data in Run 2 is 7.7 °C higher, while for the 280 °C data it is 9.7 °C lower. As a temperature offset leads to a reasonably close fit, while using the same reaction kinetics parameters, the assumption is validated, and it can be concluded that the thermocouple placement is the main source of uncertainty.

**Figure 12:**
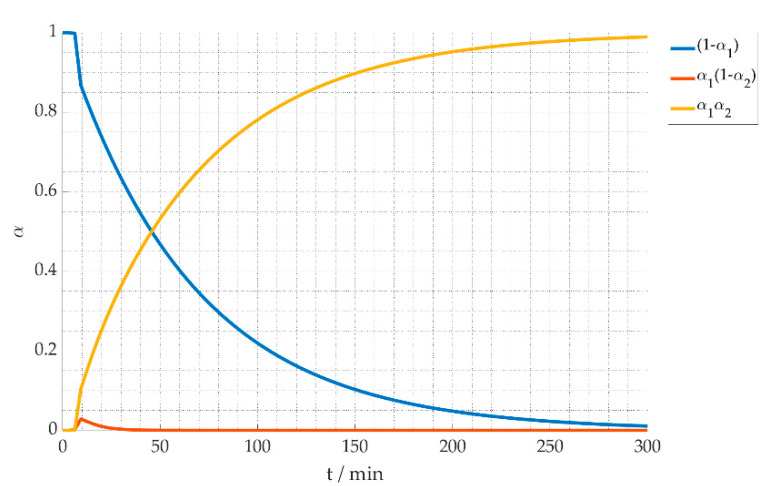
Stabilization degree α at temperature of 300 °C.

In Figure 12 the stabilization degree of the effective reaction kinetics (see Equation (15)) is plotted for the heating profile considering a heating ramp of 30 °C/min, a process temperature of 300 °C and a holding time of 300 min. It shows the decrease of the original material expressed as dielectric loss $\epsilon_{A}^{\prime \prime }$ (blue line) while intermediate (red line) and final products (yellow line) are accumulating. As soon as any intermediate product with dielectric loss $\epsilon_{B}^{\prime \prime }$ is formed, it is directly transformed into the final product $\epsilon_{C}^{\prime \prime }$. Using the dielectric properties as industrial measurement method will require calibration for different fiber qualities. Moreover, the stabilization degree needs to be calibrated according to conventionally produced material. In industrial production today, the fiber passes multiple heating zones slowly increasing temperature, and avoiding an overheating effect. In the measurements presented above, a tradeoff between ongoing reaction and temperature dependent dielectric loss had to be made. A higher heating rate was chosen to decrease the time of the chemical transformation in the heating up stage. A change in heating rate will result in a change of the dielectric properties as the reaction progresses also during the heating up stage. An optimized heating profile could be beneficial for the fiber quality, and should be investigated in future.

### 4.2. Thermal Process Model

**Figure 13:**
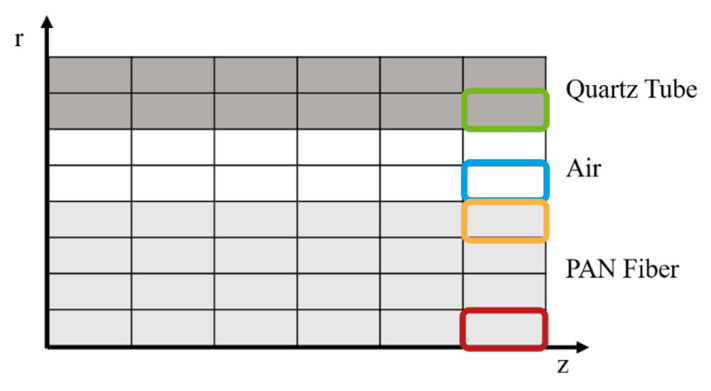
Schematic mesh with colored mesh cells that are evaluated over time in the next figures.

**Figure 14:**
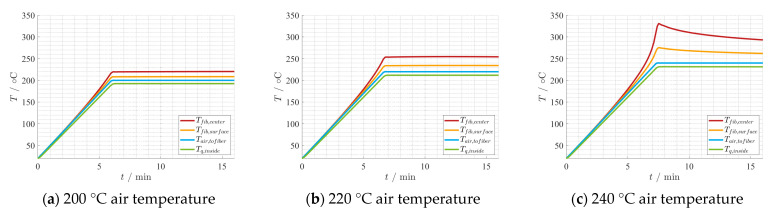
Thermal development depending on 1 W microwave power and different air temperatures overtime.

**Figure 15:**
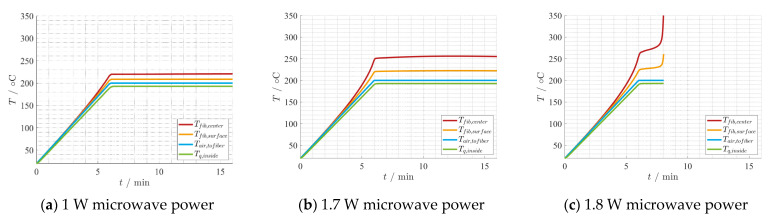
Thermal development depending on 200 °C air temperatures and different microwave power levels over time.

Together with the input parameters shown in Table 2, the findings from the reaction kinetics are implemented in the thermal model. Both the calculation of the used heat of reaction and the effective fiber properties can be found in [35]. A batch process, i.e., without fiber movement, is considered. The setting is a hybrid one, as the influence of the air flow temperature and a constant microwave power are analyzed. For the electric field strength, the simulated value from CST Microwave Studio [23] is used and adapted according to the input power level. As depicted in Figure 13, only the last mesh cell is examined over time. In Figure 14 and Figure 15, the temperature development is shown as an example for the first 10 min of holding time including the heating-up phase. Comparing Figure 14a–c the air temperature impact on the hybrid heating process becomes evident. Thus, the air temperature should always get considered in experimental and manufacturing setups. Additionally, it shows for the given specific boundary conditions, a minimum temperature is required for the chemical reaction to take place completely. In Figure 14c, the temperature of the fiber increases more than the air temperature due to the chemical reaction and the microwave heating. From Figure 15a,b, the microwave power is increased from 1 W to 1.7 W, but as result the fiber temperature increases significantly by about 30 °C. Already at a 1.8 W power input, the fiber temperature becomes unstable and the fiber is burned, as shown in Figure 15c. The simulation was stopped prematurely due to a chosen abort condition in case of temperatures over 360 °C. These simulation results shows that small variations in the process parameters can have a big impact on the process, and can lead to process instabilities and overheating of the fiber easily.

## 5. Conclusions

A test-set for high-resolution dielectric measurements, based on the cavity perturbation method in a TM_010_ mode cylindrical cavity, has been developed for the characterization of PAN fibers at 2.45 GHz. By use of systematic variation of process temperature and time for isothermal soak, for the first time a comprehensive data set of in-situ dielectric loss has been compiled during the stabilization process of PAN fibers.

To connect this data to the stabilization degree of the PAN fibers, a reaction kinetics model based on first-order reaction models was developed and combined with a simple linear dielectric mixing rule. The final model was successfully fitted to the measured time and temperature dependent dielectric data and clearly reveals the importance of accurate temperature measurement and control. The activation energies determined by the fitting routine for the combined cyclisation and dehydrogenation reaction and the subsequent oxidation reaction are comparable to the results in the literature. This validates the applicability of the model and the potential of high resolution in situ dielectric measurements for thermal analysis of these kind of chemical reactions. Furthermore, based on the derived reaction kinetics model, in situ dielectric measurements could be also used for measurement of the degree of stabilization within a PAN fiber stabilization process line.

A heat-transfer model, which includes dielectric microwave heating and the enthalpies of the exothermal stabilization reactions combined with the developed reaction kinetic model allows numerical parameter studies of a potential microwave-assisted process. By use of this model for simulation of a batch process, the influence of process parameters such as microwave power and gas temperature has been analyzed and limits for potential process instabilities and overheating have been identified. In future work, these achievements will be used to design optimized applicator geometries for a microwave-assisted continuous stabilization process using high-power solid-state microwave generators.

## Figures and Tables

**Table 1:** Fitted parameter values and confidence intervals.

Parameter	Unit	Value	Confidence Interval	
$k_{0,1}$	1/s	2.44 × 10^5^	2.36 × 10^5^	2.52 × 10^5^
$E_{a,1}$	kJ/mol	98.56	98.42	98.72
$k_{0,2}$	1/s	10.2	9.5	11
$E_{a,2}$	kJ/mol	39.2	38.86	39.52
$c_{A}$	-	1083	1068	1099
$b_{A}$	-	3689	3681	3696
$c_{B}$	-	2511	2115	2907
$b_{B}$	-	2572	2485	2658
$c_{C}$	-	14.5 × 10^8^	9.2 × 10^8^	19.8 × 10^8^
$b_{C}$	-	13.1 × 10^3^	12.9 × 10^3^	13.3 × 10^3^

**Table 2:** Model input parameter values for the temperature calculation of a batch process.

Fixed Parameter	Value	Unit
ambient air temperature	20	°C
air flow	100	l/min
cavity length	45	mm
cavity radius	45	mm
mesh cells in axial direction	30	-
fiber mesh cells in radial direction	10	-
air mesh cells in radial direction	4	-
quartz tube mesh cells in radial direction	4	-
electrical field strength from CST	12,000	V/m
heat of reaction for cyclization	−6.53 × 10^5^	J/kg
heat of reaction for dehydration	−4.933 × 10^6^	J/kg
heat of reaction for oxidation	−1.44 × 10^7^	J/kg
effective thermal conductivity of fiber, radial	0.069	W/(m·K)
effective heat capacity of fiber	1310	J/(kg·K)
emission coefficient of fiber	0.95	-
effective density of fiber	616	kg/m^3^

## Data Availability

Not applicable.
